# Small for Gestational Age and Magnesium in Cord Blood Platelets: Intrauterine Magnesium Deficiency May Induce Metabolic Syndrome in Later Life

**DOI:** 10.1155/2011/270474

**Published:** 2010-12-28

**Authors:** Junji Takaya, Kazunari Kaneko

**Affiliations:** Department of Pediatrics, Kansai Medical University, 10-15 Fumizonocho, Moriguchi, Osaka 570-8506, Japan

## Abstract

Magnesium deficiency in pregnancy frequently occurs because of inadequate or low intake of magnesium. Magnesium deficiency during pregnancy can induce not only maternal and fetal nutritional problems, but also consequences that might last in offspring throughout life. Many epidemiological studies have disclosed that small for gestational age (SGA) is associated with an increased risk of insulin resistance in adult life. We reported that intracellular magnesium of cord blood platelets is lower in SGA groups than that in appropriate for gestational age groups, suggesting that intrauterine magnesium deficiency may result in SGA. Taken together, intrauterine magnesium deficiency in the fetus may lead to or at least program insulin resistance after birth. In this review, we propose that intrauterine magnesium deficiency may induce metabolic syndrome in later life. We discuss the potential contribution of aberrant magnesium regulation to SGA and to the pathogenesis of metabolic syndrome.

## 1. Introduction

Magnesium (Mg) is an important cofactor for the enzymes that are involved in carbohydrate metabolism: an important role of Mg in insulin action has been reported [1, 2]; low serum and intracellular Mg ([Mg^2+^]_i_) concentrations are associated with insulin resistance, impaired glucose tolerance, and decreased insulin secretion [3, 4]. Furthermore, lower dietary Mg intake could cause insulin resistance both in children and adults [5, 6]. Based on these findings, we studied whether low [Mg^2+^]_i_ in the fetus would be one of the critical abnormalities associated with small for gestational age (SGA). 

In addition, several studies have shown the association of size at birth or indices in poor fetal growth with later development of metabolic syndrome and insulin resistance [7]. Hales et al. proposed that impaired glucose tolerance and type 2 diabetes might arise as a result of programming [8]. Programming is a term used to describe persistent changes in organ structure and function caused by exposure to adverse environmental influences during critical periods of development [9]. These findings have led to the “fetal origin” hypothesis, which proposes that fetal adaptation to an adverse intrauterine environment affecting fetal growth may program life-long physiological changes [10]. It has been proposed that various fetal growth patterns and intrauterine growth retardation (IUGR) arise as a result of an early stimulus or insult. Since both fetal weight and length gains are closely related, there is much overlap between SGA and IUGR. We focus on intrauterine undernutrition and SGA. SGA is defined as birth weight and/or length at least 2 standard deviations below the mean for gestational age. 

In this review, we discuss the potential contribution of aberrant Mg regulation to SGA and to the pathogenesis of metabolic syndrome later in life.

## 2. Magnesium Deficiency During Pregnancy

It is reported that the amount of maternal Mg intake is not only associated with pregnancy outcome but also with infant outcome [11]. The mean daily intake of Mg (284.3 mg/day) during pregnancy was found to be lower than recommended [12]. For example, Mg deficiency is reported to occur in 44% of pregnant Indian women [13]. Another study has demonstrated that mothers who drink water containing high amounts of Mg have a reduced risk of having very low birth weight infants (less than 1,500 g of birth weight) [14]. By the end of a normal pregnancy, the fetus is believed to have acquired approximately 28 g of calcium, 16 g of phosphorous, and 0.7 g of Mg, mostly during the third trimester, also the time when 80% of fetal accretion of Mg occurs [15]. 

The fetal circulation contains higher levels of total calcium, ionized calcium, and Mg compared to maternal blood [16]. In the placental membrane, copper and selenium share the same transport pathway along a concentration gradient in the maternal-fetal direction, while Mg and iron use predominantly an active transport pathway [17]. In fact, evidence for an active transport mechanism for Mg in the placenta has been demonstrated in cultured trophoblast cells in which low [Mg^2+^]_i_ is maintained by the function of a Na^+^/Mg^2+^ exchanger [18]. Mg levels in umbilical cord blood are the lowest in preterm low birth weight babies, followed by term low birth weight babies and are the highest in term controls (*P* < .05) [19].

Calcium and Mg have an immediate effect on placental vascular flow. Reduced placental vascular flow is at least, in part, responsible for placental insufficiency and fetal growth retardation (FGR). Calcium and Mg are cofactors of a variety of enzymes. A variety of hormones, cytokines, and growth factors produced by fetal membranes and the placenta can act locally on the myometrium [20]. The ability of the uterine artery to dilate during pregnancy may be specifically related to the upregulation of multiple pathways for the production of nitric oxide [21]. The activity of constitutive nitric oxide synthase is dependent on calcium and is inhibited by a reduction in the concentration of Mg [22].

Blood Mg levels were reportedly lower in women with severe pre-eclampsia than in healthy pregnant women (1.63 ± 0.05 mg/dL versus 1.87 ± 0.05 mg/dL; *P* < .001) [23]. Mg is widely used in obstetric practice to treat pre-eclampsia. Therapeutic levels of Mg have also been found to produce specific placental effects such as vasodilation [24]. From an *in vitro* study of human umbilical artery resistance [25], Mg sulfate was found to exert a relaxant effect on umbilical arterial tone attenuating the vasoconstrictor effect of angiotensin II and endothelin-1 in the fetal-placental vasculature. Mg sulfate used for the treatment of pre-eclampsia or hypertensive disease in pregnancy may have beneficial effects on the fetoplacental circulation. Prenatal treatment with Mg sulfate may influence calcium homeostasis and nonenzymatic antioxidant reserve in erythrocytes of preterm newborns [26].

## 3. Mg Supplements and Pregnancy Outcome

It is well known that plasma Mg falls in pregnancy because of accumulation of ion in the placenta and fetus. Mg is therefore widely given as a supplement during pregnancy, particularly in cases of preterm labor. There are several reports that oral Mg supplementation in pregnancy is safe and that it has a positive effect on fetal morbidity [27]. Patients in preterm labor have significantly depressed serum Mg levels, while in patients with pre-eclampsia, Mg levels are not significantly different from that of the controls [28]. Mg supplementation was found to decrease the rate of FGR, premature rupture of membranes, and premature delivery in risk pregnancies treated with betamimetics [29]. Oral Mg supplementation given before the 25th week of gestation is associated with a lower frequency of preterm births, a lower frequency of low birth weight, and fewer SGA infants compared with the placebo [30]. Mg intake in 513 women who were near the end of their first trimester of pregnancy was determined from records of food consumption. Mg intake correlated with weight, length, and head circumference at birth as well as length of gestation up to a threshold of around 3,200 g of birth weight [31]. On the other hand, Mg supplements (100 mg/day) taken during the second and third trimesters had no effect on the outcome of the pregnancy. The effect of Mg compared with placebo in a randomized double-blind controlled study of patients with pregnancy-induced hypertension was investigated [32]. Mg supplementation was found to be beneficial in the management of pregnancy-induced hypertension.

At present, there is insufficient high-quality evidence to show that dietary Mg supplementation during pregnancy is beneficial [30]. Any influence of Mg has been confined to the first trimester or earlier. It is important to determine the timing and dose of Mg supplementation as both factors may alter the pregnancy outcome.

## 4. The Relation of SGA to Intracellular Magnesium in Cord Blood Platelets

We have previously reported that intracellular magnesium ([Mg^2+^]_i_) is lower in children with diabetes mellitus and in children who are obese [33]. In the type 2 diabetes mellitus and obese groups, platelets responded well to insulin. Under insulin-resistant states, [Mg^2+^]_i_ decreases before the poor reactivity to insulin occurs in platelets. From these findings, we hypothesize that [Mg^2+^]_i_ is decreased earlier than when poor reactivity to insulin develops in platelets exposed to an insulin resistant environment. This suggests that low [Mg^2+^]_i_ may be an intrinsic abnormality in infants with low birth weight [33]. To test this hypothesis, we studied the relationship of [Mg^2+^]_i_ in cord blood platelets to birth weight [34]. We found that mean basal [Mg^2+^]_i_, but not plasma magnesium, is lower in SGA than in the appropriate for gestational age (AGA) groups (323 ± 162 *μ*mol/L versus 488 ± 132 *μ*mol/L, *P* = .004 ). Basal [Mg^2+^]_i_ significantly correlates with birth weight (*P* < .0001) as well as cord plasma leptin (*P* = .031) and IGF-1 (*P* < .001) [35]. [Mg^2+^]_i_ as well as leptin and IGF-1 reflects the extent of fetal growth. Also, [Mg^2+^]_i_ significantly correlates with the quantitative insulin sensitivity check index (QUICKI)) (*P* < .001). Decreased [Mg^2+^]_i_ in SGA might underlie the initial pathophysiological events that lead to insulin resistance.

Several factors are reported to affect [Mg^2+^]_i_. For example, hyperglycemia may have an effect on Mg^2+^ transport and induce a decline of [Mg^2+^]_i_. Barbagallo et al. reported that responses to hyperglycemia in vitro were blunted in adult hypertensive subjects and that these responses were closely linked to basal [Mg^2+^]_i_ levels [36]. In our study, we found no correlation between cord plasma glucose and basal [Mg^2+^]_i_ levels. In addition, we did not find any significant difference in plasma glucose levels between the SGA and AGA groups. Intrauterine glucose levels may have less of an effect on inducing SGA or low [Mg^2+^]_i_. As [Mg^2+^]_i_ plays a promotive role in fetal growth, low [Mg^2+^]_i_ may partly be responsible for SGA.

As low [Mg^2+^]_i_ is an intrinsic abnormality seen in infants with low birth weight, it is considered that fetal Mg deficiency is an important determinant of insulin resistance in later life [33]. In fact, our data was supported by a recent animal study demonstrating that maternal Mg restriction irreversibly increases body fat and induces insulin resistance in pups by 6 months of age [37].

## 5. Fetal Programming

The fetal origin hypothesis by Barker et al. [8–10] states that fetal undernutrition in middle to late gestation leads to disproportionate fetal growth programs and later metabolic diseases. Fetal programming is a phenomenon in which alterations in fetal growth and development in response to the prenatal environment have long-term or permanent effects. This theory was further established in the category of developmental origins of health and disease (DOHaD) [38]. The mechanisms thought to be responsible for fetal programming include the direct effects on cell number, altered stem cell function, and the resetting of regulatory hormonal axes: hypothalamic-pituitary-adrenal axis [39, 40] and growth hormone insulin-like growth factor axis [41]. One of the underlying mechanisms for the postulated early-life programming is that of epigenetics. Altered epigenetic regulation of genes in phenotype induction could possibly give rise to interventions that modify long-term disease risk associated with unbalanced nutrition in early life [42]. Venu et al. reported that fasting insulin and Homeostasis Model Assessment-Insulin Resistance (HOMA-IR) increased in offspring from Mg-restricted dams at 6 months of age [37]. Maternal and postnatal Mg status is important in the long-term programming of body adiposity and insulin secretion in rat offspring [43].

Although low birth weight and poor prenatal nutrition are strongly associated with metabolic syndrome in later life, postnatal catch-up growth was recently considered to also be a pivotal element associated with the development of various pathological conditions [44]. The concept of a sensitive or crucial period that operates to cause long-term changes in development and adverse outcomes later in life is an intriguing one and should be the focus of more studies in the future.

## 6. Conclusion

Birth weight is only a crude index of early growth and indicates nothing about the success of a fetus in achieving its growth potential. [Mg^2+^]_i_ may be a marker of early growth restriction, which may be of future diagnostic use as an early predictor of adult diseases. Low [Mg^2+^]_i_, which may represent the prenatal programming of insulin resistance, has lifelong effects on metabolic regulation. A biological interpretation of the association between birth size and risk of insulin-resistant diseases should emphasize the possible underlying roles of [Mg^2+^]_i_.

## Abbreviations

AGA: Appropriate for gestational age
FGR: Fatal growth retardation
Mg: Magnesium
[Mg^2+^]_i_: Intracellular magnesium
SGA: Small for gestational age.
